# Prostaglandin E_2_ stimulates normal bronchial epithelial cell growth through induction of c-Jun and PDK1, a kinase implicated in oncogenesis

**DOI:** 10.1186/s12931-015-0309-0

**Published:** 2015-12-18

**Authors:** Yu Fan, Ye Wang, Ke Wang

**Affiliations:** Department of Respiratory Medicine, West China Hospital, Sichuan University, Chengdu, Sichuan Province 610041 China; Department of Radiotherapy, Sichuan Cancer Hospital, Chengdu, Sichuan Province 610041 China

**Keywords:** Prostaglandin E2, PDK1, c-Jun, Human normal bronchial epithelial cells

## Abstract

**Background:**

Cyclooxygenase-2-derived prostaglandin E_2_ (PGE_2_), a bioactive eicosanoid, has been implicated in many biological processes including reproduction, inflammation and tumor growth. We previously showed that PGE_2_ stimulated lung cancer cell growth and progression through PGE_2_ receptor EP2/EP4-mediated kinase signaling pathways. However, the role of PGE_2_ in controlling lung airway epithelial cell phenotype remains unknown. We evaluated the effects of c-Jun and 3-phosphoinositede dependent protein kinase-1 (PDK1) in mediating epithelial cell hyperplasia induced by PGE_2_.

**Method:**

The bronchial epithelial cell lines BEAS-2B and HBEc14-KT were cultured and then treated with PGE_2_. PDK1 small interfering RNA (siRNA) and a PDK1 inhibitor, an antagonist of the PGE_2_ receptor subtype EP4 and EP4 siRNA, c-Jun siRNA, and overexpressions of c-Jun and PDK1 have been used to evaluate the effects on cell proliferation.

**Results:**

We demonstrated that PGE_2_ increased normal bronchial epithelial cell proliferation through induction of PDK1, an ankyrin repeat-containing Ser/Thr kinase implicated in the induction of apoptosis and the suppression of tumor growth. PDK1 siRNA and a PDK1 inhibitor blocked the effects of PGE_2_ on normal cell growth. The PGE_2_-induced PDK1 expression was blocked by an antagonist of the PGE_2_ receptor subtype EP4 and by EP4 siRNA. In addition, we showed that induction of PDK1 by PGE_2_ was associated with induction of the transcription factor, c-Jun protein. Silencing of c-Jun using siRNA and point mutations of c-Jun sites in the PDK1 gene promoter resulted in blockade of PDK1 expression and promoter activity induced by PGE_2_. In contrast, overexpression of c-Jun induced PDK1 gene promoter activity and expression followed increased cell proliferation.

**Conclusion:**

PGE_2_ increases normal bronchial epithelial cell proliferation through increased PDK1 gene expression that is dependent on EP4 and induction of c-Jun. Therewith, our data suggest a new role of c-Jun and PDK1 in mediating epithelial cell hyperplasia induced by PGE_2_.

## Background

Cancer claims over half a million lives in the United States annually, and lung cancer is the number one cause of cancer death in both men and women. An estimated 226,160 new cases of lung cancer was diagnosed in 2012 in the United States alone, and 160,340 lung cancer deaths are estimated to occur [1]. Chronic inflammation has been associated with an increased risk of cancer. Protein levels of cyclooxygenase-2 (COX-2), a mediator of inflammation, were reported elevated in several cancer types, including colorectal, prostate, and lung cancers, and suppression of either COX-2 expression or COX-2 activation, is being considered for cancer prevention and therapy [2]. Of the five prostaglandins (PG) produced by COX-2, PGE_2_ appeared to play essential roles in tumor cell proliferation, invasion, angiogenesis, and immunosuppression [3–5]. Four receptor subtypes are known to bind PGE_2_, and they are named EP1-4. These receptors have also been implicated in tumor cell growth and progression [6, 7]. PGE_2_ is increased in patients with chronic obstructive pulmonary disease also showing a higher incidence of lung cancer [8].

Three-phosphoinositede dependent protein kinase-1 (PDK1) is a master kinase, which is crucial for the activation of AKT / protein kinase B (PKB) and many other AGC kinases including protein kinase C (PKC), S6 protein kinase (S6K), and serum-and glucocorticoid-induced protein kinase (SGK) [9]. As an upstream regulator of AKT, PDK1 signaling is thought to play a key role in cancer cell growth, survival and tumor angiogenesis [10, 11]. Studies have shown high levels of activated PDK1 in a large percentage of common tumor types, including melanoma, breast, lung, gastric, prostate, hematological, and ovarian cancers [12]. When activated in tumor cells, Akt also has multiple effects that promote disease progression, including suppression of apoptosis and stimulation of tumor cell proliferation, metabolism, and angiogenesis [13–15]. Thus, the PDK1/Akt signaling pathway represents an attractive target for the development of small molecule inhibitors that may be useful in the treatment of cancer. However, the effects of PGE_2_ on human bronchial epithelial cells are not clear. Here, we explore the effects of PGE_2_ on human bronchial epithelial cell proliferation and the intracellular signals involved in this process. Our studies show that PGE_2_ stimulates human bronchial epithelial cell proliferation through the EP4 receptor and activation of c-Jun, which increases the expression of PDK1.

## Methods

### Cell culture and chemicals

The human bronchial epithelial cell lines BEAS-2B and HBEc14-KT were obtained from John Minna (University of Texas Southwestern Medical Center, Dallas, TX, USA). They were maintained in KERATINOCYTE-SFM medium (Invitrogen Corporation, Carlsbad, CA, USA) supplemented with human recombinant epidermal growth factor 1–53 (EGF 1–53) and bovine pituitary extract (BPE). Cells were plated into six-well culture plates at an initial seeding density of 5 × 10^4^ cells per well. The plates were incubated in a humidified atmosphere of 5 % CO_2_ in air at 37 °C. Lipofectamine 2000 reagent was purchased from Invitrogen. The CellTiter-Glo Luminescent Cell Viability Assay kit was purchased from Promega. Polyclonal antibodies against PDK1 and phosphorylated PDK1 (ser 241) were purchased from Cell Signaling. The polyclonal antibody against c-Jun was purchased from Santa Cruz Biotechnology, Inc. Polyclonal antibody against EP4, a 16, 16-Dimethyl-PGE_2_ (dmPGE2) solution in methyl acetate and AH23848, OSU-03012 were purchased from Cayman Chemical Co.

### Reverse transcription and real time PCR

Real time PCR was performed to assess whether PDK1 expression was modulated by PGE_2_. Total RNA was isolated using the RNA Bee kit (Qiagen, Inc., Valencia, CA) as per manufacturer’s instructions. One μg of total RNA was reverse transcribed as per protocol using TaqMan® RT reagents (Applied Biosystems) at 37 °C for 120 min followed by 25 °C for 10 min.

Forty ng of cDNA per reaction were used in the real time PCR using the ABI Prism® 7900HT Sequence Detection System (Foster City, CA). In the presence of AmpliTaq Gold DNA polymerase (ABI Biosystems, Foster City, CA), the reaction was incubated for 2 min at 50 °C followed by 10 min at 95 °C. Then the reaction was run for 40 cycles at 15 s, 95 °C and 1 min, 60 °C per cycle (Table 1). Assays-on-Demand™ primers and probes specific for the oxytocin receptor (Applied Biosystems; ID number Hs00168573-m1) were used in the PCR. GAPDH was measured and used to normalize all samples using the ΔΔCT method (Applied Biosystems). Gene expression of PDK1 is expressed relative to GAPDH and untreated samples in each stimulation study, respectively. At least three replicates were run for each condition.

**Table 1:** Reverse transcription and real time PCR primer

	Stage	Temp	Time
Reverse transcription	hold	37 °C	120 min
	hold	25 °C	10 min
PCR	hold	50 °C	2 min
	denature	95 °C	15 s
	anneal/extend	60 °C	1 min
	hold cycle (40 cycles)	95 °C	10 min
Primer	Sense 5′ AGATGAGTGACCGAGGAG		
	Antisense 3′ TATACACTGGGAGTCTTTCT		

### Western blot analysis

The procedure was performed as previously described [16]. Protein concentrations were determined by the Bio-Rad protein assay. 5 μg protein from whole-cell lysates were solubilized in 5× SDS sample buffer and separated on SDS 10 % polyacrylamide gels. Blots were incubated with antibodies against c-Jun, PDK1, EP4, GAPDH, and phosphorylated PDK1 (c-Jun, PDK1, pPDK1 and EP4 in the concentrations of 1:1,000 for 2 h; GAPDH in the concentration of 1:10000 for 1 h) at room temperature. After washing several times, the blots were followed by incubation with a secondary goat antibody raised against rabbit IgG conjugated to horseradish peroxidase (1:2,000; Santa Cruz). The blots were washed, transferred to fresh chemiluminescence solution (Amersham) for about 1–2 min, and exposed to X-ray film. In controls, the primary antibodies were omitted or replaced with a control rabbit IgG.

### Treatments with EP4, PDK1, and c-Jun small interfering RNA and expression vector

The EP4, PDK1, c-Jun siRNA and control nonspecific siRNA oligonucleotides were purchased from Santa Cruz Biotechnology. pWZL-Neo-Myr- Flag PDK1 and pWZL-Neo-Myr-Flag-DEST were purchased from Addgene Inc. The pGME4 c-Jun vector and pGME4 were provided by Dr. Tom Curran (Children’s Hospital of Philadelphia, University of Pennsyvania, USA). For the transfection procedure, cells were grown to 60 % confluence, and EP4, c-Jun and PDK1 siRNAs,control siRNA,and expression vector were transfected using the lipofectamine 2000 reagent according to the manufacturer’s instructions. Briefly, the lipofectamine reagent was incubated with serum-free medium for 5 min. Subsequently, a mixture of respective siRNA was added. After incubation for 20 min at room temperature, the mixture was diluted with medium and added to each well. The final concentration of siRNAs in each well was 100 nmol/L. After culturing for 30 h, cells were washed and resuspended in new culture medium in the presence or absence of dmPGE_2_ for Western blot and cell growth and gel mobility shift assays.

### Transient transfection assay

The human PDK1 promoter ligated to the luciferase reporter gene were a gift from Drs. Michalik and Desvergne (Center for Integrative Genomics, University of Lausanne, Lausanne, Switzerland) and have been reported previously [17]. The PDK1 promoter construct contains ∼ 663 bp of the human PDK1 promoter connected to the pGL_2_ vector. Briefly, normal bronchial epithelial cells were seeded at a density of 5 × 10^5^ per well in 24-well dishes and grown to 50–60 % confluence. For each well, 0.4 μg of the above PDK1 plasmid DNA constructs, with or without 0.5 μg of the internal control phRL-TK Synthetic *Renilla* Luciferase Reporter Vector, were co-transfected into the cells using lipofectamine 2000 reagent . After 24 h of incubation, cells were treated with or without dmPGE_2_ for 4 h. The preparation of cell extracts and measurement of luciferase activities were carried out using the Dual-Luciferase Reporter kit according to recommendations by the manufacturer (Promega). The assays for firefly luciferase activity and *Renilla* luciferase activity were performed sequentially in a Labsystems Luminoskan Ascent luminometer equipped with dual injectors. Changes in firefly luciferase activity were calculated and plotted after normalization with changes in *Renilla* luciferase activity within the same sample.

### Cell viability assay

Normal bronchial epithelial cells were plated at the indicated densities (2 × 10^3^ cells/well) in 96-well multiwell culture plates (Costar). Cells were treated with inhibitor or antagonist for 2 h before exposure of the cells to PGE_2_ in the culture medium (containing 10 % FBS). In separate experiments, cells were transfected with control, PDK1, EP4 or c-Jun siRNAs or expression vector for 40 h before exposure to PGE_2_ for up to 4 days. Cell proliferation was evaluated using the CellTiter-Glo Luminescent Cell Viability Assay, a homogenous method of determining number of viable cells in culture based on quantitation of the ATP present which signals the presence of metabolically active cells.

### Statistical analysis

All experiments were repeated a minimum of three times. All data from western blot analysis, real-time PCR, and luciferase assays were expressed as mean ± SD. In cell viability assay, the bar graphs represented the mean ± s.d. of relative cell viability compared to the control group of at least three independent experiments. In western blot analysis, the optical densities (OD) of pPDK1, PDK1, EP4 and C-Jun were normalized to the OD of GAPDH in the same membrane. The data represented the mean ± s.d. of relative OD compared to the control group of at least three independent experiments with three samples in each. In transient transfection assay, the bar graphs represent the mean ± s.d. of relative luciferase activities compared to the control group of at least three independent experiments. One-way anova analyses followed by the Least Significant Difference (LSD) test were performed. Asterisks showed in the figures indicate significant differences of experimental groups in comparison with the corresponding control condition. *P*-values <0.05 were considered statistically significant.

## Results

### PGE_2_ increases cell proliferation and phosphorylation and expression of PDK1 in HBEc14-KT and BEAS2B cells

**Fig. 1:**
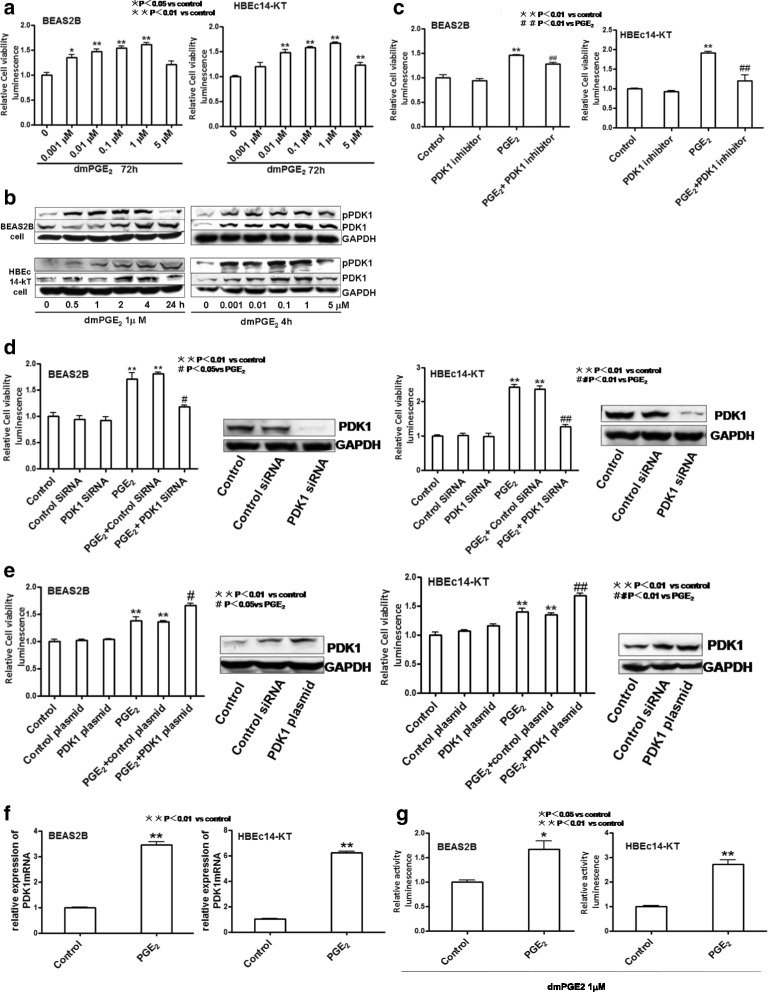
The effect of dmPGE_2_ on human bronchial epithelial cell proliferation and induction of expression and activation of PDK1. **a** Effects on cell proliferation. **b** dmPGE_2_ increases the phosphorylation and expression of PDK1 in a time- and dose-dependent manner in BEAS2B and HBEc14-KT cells. **c** PDK1 inhibitor OSU-03012 decreases cell proliferation induced by dmPGE_2_ in normal bronchial epithelial cells. **d** PDK1 siRNA decreases the cell proliferation induced by dmPGE_2_ in normal bronchial epithelial cells. **e** PDK1 plasmid increases the cell proliferation induced by dmPGE_2_ in normal bronchial epithelial cells. **f** Effect on mRNA expression. **g** Effect on PDK1 promoter activity

PGE_2_ has been shown to stimulate lung cancer cell proliferation [18]. To examine the effects of PGE_2_ on human bronchial epithelial cell proliferation, HBEc 14-KT and BEAS2B cells were treated with increased concentrations of dmPGE_2_ for 72 h. As shown in Fig. 1a, PGE_2_ stimulates normal bronchial epitheial cell proliferation in a dose-dependent manner with maximal effect at a concentration of 1 μM, compared to the control group (1.604 ± 0.046 vs 1.000 ± 0.046 in BEAS2B, *P* <0.01; 1.665 ± 0.023 vs 1.000 ± 0.017 in HBEc14-KT, *P* <0.01). Exposure to PGE_2_ enhances the phosphorylation and expression of PDK1 in a dose-dependent and time-dependent manner with maximal effect at a concentration of 1 μM at 2–4 h (Fig. 1b). pPDK1 and PDK1 reached their peaks at 4 h with the concentration of 1 μM of dmPGE_2_, compared to the control group (3.414 ± 0.243 vs 1.000 ± 0.135 and 1.512 ± 0.087 vs 1.001 ± 0.129 in BEAS2B, *P* <0.01, *P* <0.05; 1.373 ± 0.092 vs 1.000 ± 0.142 and 1.415 ± 0.726 vs 1.000 ± 0.130 in HBEc14-KT, *P* <0.01, *P* < 0.01). pPDK1 and PDK1 reached their peaks with the concentration of 1 μM of dmPGE_2_ after incubation for 4 h, compared to the control group (2.812 ± 0.317 vs 1.000 ± 0.216 and 5.214 ± 0.478 vs 1.000 ± 0.198 in BEAS2B, *P* <0.01, *P* <0.01; 2.754 ± 0.139 vs 1.000 ± 0.141 and 2.351 ± 0.286 vs 1.000 ± 0.127 in HBEc14-KT, *P* <0.01, *P* <0.01). To evaluate the role of PDK1 in PGE_2_-induced cell proliferation, PDK1 was silenced with siRNA or pre-treated with PDK1 inhibitor OSU-03012 (2 μM) in the cell lines (Fig. 1c and d). PDK1 inhibitor (OSU-03012) decreased cell proliferation induced by treatment with PGE_2_ (1.279 ± 0.030 vs 1.463 ± 0.005 in BEAS2B, *P* <0.01; 1.211 ± 0.142 vs 1.918 ± 0.038 in HBEc14-KT, *P* <0.01). PDK1 siRNA decreased cell proliferation induced by treatment with PGE_2_ (1.177 ± 0.038 vs 1.708 ± 0.127 in BEAS2B, *P* <0.05; 1.272 ± 0.052 vs 2.428 ± 0.073 in HBEc14-KT, *P* <0.01). In contrast, transfection of cells with PDK1 plasmid increases cell proliferation induced by PGE_2_ (1.653 ± 0.042 vs 1.381 ± 0.067 in BEAS2B, *P* <0.05; 1.681 ± 0.033 vs 1.395 ± 0.057 in HBEc14-KT, *P* <0.01) (Fig. 1e).

Having established a role for PDK1 in PGE_2_-stimulated normal bronchial epithelial cell proliferation, we examined whether the effects of PGE_2_ on PDK1 expression occur at the transcriptional level. We found that PGE_2_ induces the expression of PDK1 mRNA (3.462 ± 0.103 vs 1.000 ± 0.020 in BEAS2B, *P* <0.01; 6.218 ± 0.138 vs 1.008 ± 0.069 in HBEc14-KT, *P* <0.01) (Fig. 1f) and activity of PDK1 gene promoter (1.666 ± 0.177 vs 1.000 ± 0.039 in BEAS2B, *P* <0.05; 2.714 ± 0.187 vs 1.000 ± 0.043 in HBEc14-KT, *P* <0.01) (Fig. 1g). We therefore conclude that PGE_2_ increases cell proliferation in normal bronchial epithelial cells by activation of the PDK1.

### EP4 mediates the cell proliferation and increased expression and activation of PDK1 induced by PGE_2_ in HBEc14-KT and BEAS2B cells

**Fig. 2:**
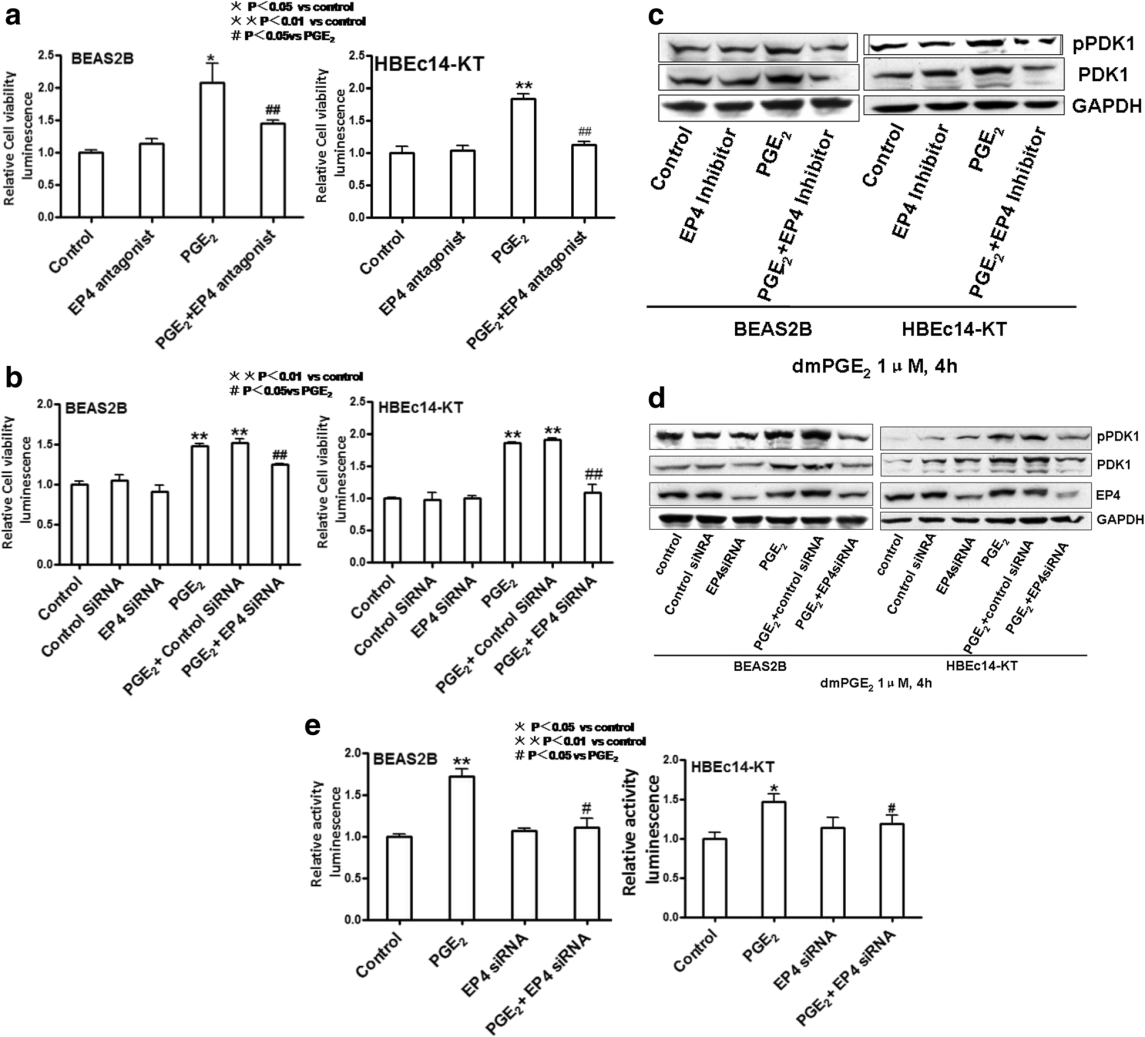
EP4 Mediates the Cell Proliferation and increased expression and activation of PDK1 induced by PGE_2_ in HBEc14-KT and BEAS2B cells. **a** EP4 antagonist AH23848 decreased the cell proliferation induced by dmPGE_2_ in normal bronchial epithelial cells. **b** EP4 siRNA decreases the cell proliferation induced by dmPGE_2_ in normal bronchial epithelial cells. **c** EP4 antagonist AH23848 decreased the phosphorylation and expression of PDK1 induced by dmPGE_2_ in BEAS2B and HBEc14-KT cells. **d** EP4 siRNA decreased the phosphorylation and expression of PDK1 induced by dmPGE_2_ in BEAS2B and HBEc14-KT cells. **e** Effect on PDK1 promoter activity

PGE_2_ binds to and activates four distinct receptor subtypes named EP1-4. EP4 has been shown to a target molecule in cancer cell proliferation [19]. To investigate the mechanisms involved in PGE_2_-mediated bronchial epithelial cell proliferation, we transfected cells with EP4 siRNA or pre-treated cells with the EP4 antagonist AH23848 (10 μM). After pre-treatment with EP4 siRNA or antagonist, cell proliferations induced by PGE_2_ decreased (Fig. 2a and b). EP4 antagonist AH23848 decreased cell proliferation induced by PGE_2_ (1.456 ± 0.046 vs 2.078 ± 0.299 in BEAS2B, *P* <0.01; 1.131 ± 0.052 vs 1.838 ± 0.074 in HBEc14-KT, *P* <0.01). EP4 siRNA decreased cell proliferation induced by treatment with PGE_2_ (1.243 ± 0.018 vs 1.480 ± 0.029 in BEAS2B, *P* <0.05; 1.086 ± 0.124 vs 1.866 ± 0.015 in HBEc14-KT, *P* <0.01). This effect was associated with inhibition of phosphorylation and expression of PDK1 (Fig. 2c and d). EP4 antagonist AH23848 decreased the phosphorylation (1.213 ± 0.013 vs 1.457 ± 0.021 in BEAS2B, *P* <0.05; 1.198 ± 0.019 vs 1.562 ± 0.029 in HBEc14-KT, *P* <0.05) and expression of PDK1 (1.012 ± 0.008 vs 1.452 ± 0.025 in BEAS2B, *P* <0.05; 1.213 ± 0.019 vs 1.483 ± 0.024 in HBEc14-KT, *P* <0.05) induced by dmPGE_2_. EP4 siRNA decreased the phosphorylation (0.987 ± 0.019 vs 1.621 ± 0.038 in BEAS2B, *P* <0.05; 1.242 ± 0.022 vs 1.765 ± 0.031 in HBEc14-KT, *P* <0.05) and expression of PDK1 (1.056 ± 0.011 vs 1.513 ± 0.018 in BEAS2B, *P* <0.05; 1.356 ± 0.028 vs 2.107 ± 0.039 in HBEc14-KT, *P* <0.05) induced by dmPGE_2_. It also decreased PDK1 gene promoter activity (1.104 ± 0.113 vs 1.716 ± 0.088 in BEAS2B, *P* <0.05; 1.187 ± 0.111 vs 1.468 ± 0.101 in HBEc14-KT, *P* <0.01) induced by dmPGE_2_ (Fig. 2e). So we conclude that EP4 mediates cell proliferation induced by PGE2 in normal bronchial epithelial cells.

### The role of transcription factor c-Jun in PGE_2_ induction of PDK1 and cell growth

**Fig. 3:**
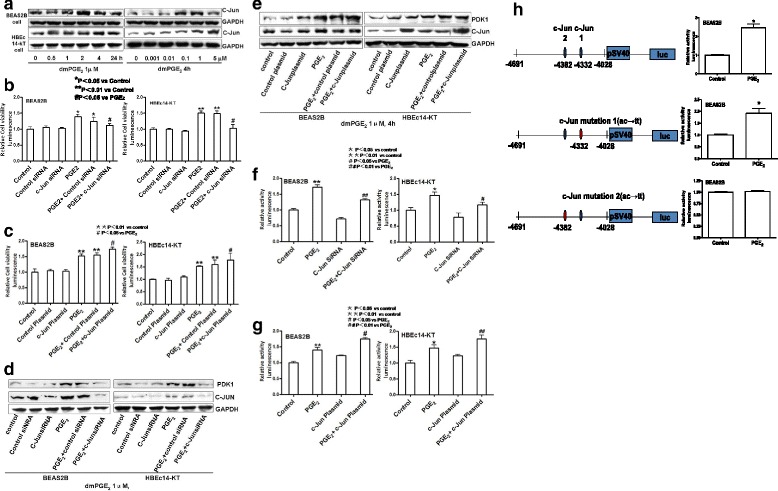
c-Jun Mediates the Cell Proliferation and increased expression and activation of PDK1 induced by PGE_2_ in HBEc14-KT and BEAS2B cells. **a** dmPGE_2_ increases expression of c-Jun in a time- and dose-dependent manner in BEAS2B and HBEc14-KT cells. **b** c-Jun siRNA decreases the cell proliferation induced by dmPGE_2_ in normal bronchial epithelial cells. **c** c-Jun plasmid increases the cell proliferation induced by dmPGE_2_ in normal bronchial epithelial cells. **d** c-Jun siRNA decreases expression of PDK1 induced by dmPGE_2_ in BEAS2B and HBEc14-KT cells. **e** c-Jun plasmid increases expression of PDK1 induced by dmPGE_2_ in BEAS2B and HBEc14-KT cells. **f** Effect of c-Jun siRNA on PDK1 promoter activity. **g** Effect of c-Jun plasmid on PDK1 promoter activity. **h** Effects of c-Jun point-moutations on PDK1 promoter activity

The PDK1 gene promoter contains multiple transcription factor-binding sites, including two sites for c-Jun. Then we explored the role of c-Jun in mediating PGE_2_-induced PDK1 expression in normal bronchial epithelial cells. PGE_2_ induces the expression of c-Jun protein in a time-dependent and dose-dependent manner (Fig. 3a). C-Jun reached its peaks at 2 h in BEAS2B cells and 4 h in HBEc14-KT cells with the concentration of 1 μM of dmPGE_2_, compared to the control group (2.561 ± 0.189 vs 1.000 ± 0.063 in BEAS2B, *P* <0.01; 1.347 ± 0.132 in HBEc14-KT, *P* <0.01). c-Jun reached its peak with the concentration of 1 μM of dmPGE_2_ after incubation for 4 h, compared to the control group (1.499 ± 0.108 vs 1.001 ± 0.063 in BEAS2B, *P* <0.01; 1.536 ± 0.174 vs 1.000 ± 0.024 in HBEc14-KT, *P* <0.01). Silencing of c-Jun with its siRNA decreases cell proliferation (1.115 ± 0.054 vs 1.386 ± 0.072 in BEAS2B, *P* <0.05; 1.030 ± 0.104 vs 1.495 ± 0.072 in HBEc14-KT, *P* <0.01) (Fig. 3b) and the expression of PDK1 induced by PGE_2_ (1.105 ± 0.011 vs 2.534 ± 0.029 in BEAS2B, *P* <0.01; 1.157 ± 0.021 vs 1.892 ± 0.027 in HBEc14-KT, *P* <0.01) induced by dmPGE_2_ (Fig. 3d). Transfection with c-Jun plasmid increases the cell proliferation (1.741 ± 0.051 vs 1.518 ± 0.059 in BEAS2B, *P* <0.05; 1.772 ± 0.258 vs 1.521 ± 0.027 in HBEc14-KT, *P* <0.05) (Fig. 3c) and the expression of PDK1 induced by PGE_2_ (2.278 ± 0.134 vs 1.196 ± 0.095 in BEAS2B, *P* <0.05; 2.453 ± 0.141 vs 2.056 ± 0.163 in HBEc14-KT, *P* <0.05) (Fig. 3e).

Having shown the important role of PDK1 in PGE_2_-related NSCLC proliferation, we further investigated whether the PGE_2_-mediated up-regulation of PDK1 reflected transactivation of the gene. To this end, we performed transient transcription experiments using human PDK1 promoter-reporter constructs connected to a luciferase reporter gene and found that PGE_2_ increased PDK1 promoter activity. The induction of PDK1 promoter activity by PGE_2_ was inhibited by c-Jun siRNA (1.319 ± 0.028 vs 1.716 ± 0.069 in BEAS2B, *P* <0.01; 1.171 ± 0.071 vs 1.468 ± 0.101 in HBEc14-KT, *P* <0.05) (Fig. 3f). Exogenous overexpression of c-Jun enhanced the PDK1 promoter activity (1.753 ± 0.037 vs 1.401 ± 0.075 in BEAS2B, *P* <0.05; 1.762 ± 0.117 vs 1.468 ± 0.101 in HBEc14-KT, *P* <0.01) (Fig. 3g). The induction of PDK1 gene promoter activity by PGE_2_ was abrogated when the one of c-Jun sites at -4382 bp was mutated in the PDK1 gene promoter, suggesting a critical role for c-Jun in mediating the effect of PGE_2_ on PDK1 gene promoter activity (Fig. 3h). dmPGE_2_ increased PDK1 promoter activity (2.432 ± 0.358 vs 1.000 ± 0.039 in BEAS2B, *P* <0.05); point mutation of c-Jun with ac to tt at −4028 site could not derease PDK1 promoter activity (1.9832 ± 0. vs 1.000 ± 0.024 in BEAS2B, *P* <0.05); point mutation of c-Jun site with ac to tt at -4382 bp dereased PDK1 promoter activity (1.010 ± 0.011 vs 1.000 ± 0.039 in BEAS2B, *P* <0.05);

## Discussion

The transformation is driven by both endogenous and exogenous factors including chemical agents that induce the activation of cancer-promoting genes. Accumulating epidemiologic and clinical data provide a strong link between inflammation and cancer initiation and progression [20, 21], but the molecular inflammatory determinants remain to be established. COX-2 derived PGE_2_ is a proinflammatory bioactive lipid and is the major prostaglandin produced in many human solid tumors, including cancer of the colon [22], stomach [23], breast [24] and lung [25]. PGE_2_ and its receptors play an essential role in promoting cancer progression. For example, PGE_2_ significantly enhanced carcinogen induced colon tumor incidence and multiplicity in rats. PGE_2_ accelerates intestinal adenoma growth in *Apc*^*Min*^ mice [26]. However, the effects of PGE_2_ on human bronchial epithelial cells are not clear.. Therefore, we will focus on PGE2 and its receptors and its downstream targets in human bronchial epithelial cells, and explore the potential mechanism. This is the first study on the effects of PGE_2_ in inducing the activation of cancer-promoting genes in normal bronchial epithelial cells. In concordance with studies performed in tumor cells before, we demonstrate PGE_2_ increases normal bronchial epithelial cell proliferation, through increased PDK1 gene expression that is dependent on EP4 and induction of c-Jun. It unveils a novel role of c-Jun and PDK1 in mediating epithelial cell hyperplasia induced by PGE_2_.

Three-phosphoinositide-dependent protein kinase-1 (PDK1) is a major mediator of cellular signaling between phosphoinositide-3 kinase and various intracellular serine/threonine kinases, including PKB, p70 ribosomal S6 kinase, serum and glucocorticoid-inducible kinase, and protein kinase. PDK1 is able to phosphorylate Thr-308 on PKBα [27, 28], which has been shown to play a crucial role in normal and pathophysical conditions. Activation of PDK1 has been shown to regulate cell survival and growth, cell cycle progression, gene expression and differention [9, 29]. Autophosphorylation and growth-factor-induced phosphorylation in its activation loop is required for PDK1 activity [30]. S241A mutation abolished PDK1 catalytic activity completely, which suggests phosphorylation at Ser-241 is important for PDK1 signaling [31]. There is increasing evidence that PDK1 is involved in cancer progression and invasion. Tissue microarray analysis of human invasive breast cancer has revealed that phosphorylation of PDK1 on Ser-241 was strongly enhanced in 90 % of the samples tested [11]. Immunohistochemical analysis using anti-phospho-Tyr-9 antibodies demonstrated that the level of Tyr-9 phosphorylation is increased markly in diseased lung, liver, colon, and breast tissue compared to normal tissue [12]. Studies have shown that angiotension-II-induced focal adhesion formation is inhibited by infection with Adeno-PDK1-Y9F via paxillin [10]. This regulation of focal adhesion suggests that PDK1 participates in integrating signals that control cell growth, apoptosis and migration. PDK1 gene has been assoiated with poor differentiation of late stage lung cancer [32]. PDK1 was shown capable of augmenting tumorigenesis in tissues harboring ERBB2 amplifications [33], PTEN deletions [34], and mutations in the catalytic subunit of phosphoinositide 3-kinase (PIK3CA) [35]. Inhibition of PDK1 is therefore expected to attenuate tumors associated with deregulated PIK3CA/PTEN signaling. Indeed, hypomorphic mutation of PDK1 in Pten ^+/−^ mice delays the onset of tumorigenesis, and small molecule inhibitors of PDK1 inhibit tumor xenografts and lung colonization [36]. Further, PDK1 inactivation effectively attenuated the development of *Kras* oncogene-driven pancreatic cancer, but not NSCLC, further supporting the importance of PDK1 in tumor development, albeit, in select cancer types [37]. Here, we found that PGE2 increased the expression of PDK1 in bronchial epithelial cells. Overexpression of PDK1 enhanced cell proliferation, while cells stimulated with PDK1 inhibitor or siRNA reduced cell growth. These findings provides the first genetic evidence that PDK1 may also play an essential role in abnormally enhanced cell proliferation induced by PGE2 in bronchial epithelial cells, which was convinced in tumor cells in previous studies. Consistent with former studies, PDK1 inactivations by small molecule inhibitor of PDK1 or siRNA effectively attenuated cell proliferation in bronchial epithelial cells, supporting the potential importance of PDK1 in NSCLC development. PDK1 also plays a crucial role in metastasis. This kinase mediates mammary epithelial cell growth and invasion in the transformed phenotype, in part, by membrane type 1 matrix metalloproteinase (MMP) induction, which in turn activates MMP-2 and modulates the extracellular matrix proteins decorin and collagen [11]. Knockdown of PDK1 inhibits spontaneous migration and epidermal-growth-factor-induced chemotaxis in breast cancer cells. In severe combined immunodeficiency mice, PDK1-depleted human breast cancer cells form tumors more slowly and are defective in extravasation to the lungs after intravenous injection [38]. These results indicate that PDK1 plays an important role in regulating malignancy in breast cancer cells. Moreover, reducing PDK1 expression in PTEN ^+/−^ mice protects these animals from developing a wide range of tumors [36], thereby providing genetic evidence that PDK1 is a key effector in mediating neoplasia that result from loss of PTEN. In addition, PDK1 is an upstream kinase of AKT, which regulates activities of AKT, in turn, mediates the regulation of tumor cell growth, metastasis, and angiogenesis [39]. All these results validate PDK1 is a promising anticancer target for the prevention of tumors.

PGE2 exerts its cellular effects by binding to its cognate receptors (EP1-4) that belong to the family of seven transmembrane G protein coupled rhodopsin-type receptors [40]. The central role of PGE2 in tumor-genesis has been further confirmed through homozygous deletion of its receptors. Mice with homozygous deletion in EP1 and EP4 receptors, but not EP3, were partially resistant to colon carcinogen mediated induction of aberrant crypt foci [41, 42]. EP2 disruption decreases the number and size of intestinal polyps in APC^△716^ knockout mice [43]. In addition to colorectal cancer, it has been shown that EP1, 2, and 4 receptors were elevated whereas EP3 receptor levels were decreased in mammary tumors in COX-2-MMTV mice [44]. It was showed that PGE_2_ promotes lung cancer cell migration via EP4-betaArrestin1-c-Src pathway [45]. In this study, blockade of EP4 by antagonists or by EP4 siRNA resulted in reduced PDK1 protein expression and activity of PDK1 promoter, suggesting that EP4 mediates the effect of PGE2 on PDK1 expression in normal bronchial epithelial cells. The EP4 receptor can activate several pathways that involve cell proliferation, adhesion, invasion and migration. These main molecules include G protein, adenylyl cyclase, protein kinase A, exchange protein directly activated by cAMP, phosphodiesterase, phosphatidylinositol 3-Kinase, extracellular signal-regulated kinase. Most recently, we found that the EP4 receptor could increase the expression of JNK [16], which has been found to play a pivotal role in activating transcription factors (including c-Jun) that increase cellular growth and tumor formation in NSCLC cells. We also showed that activation of PI3-K, PKA, and JNK induced by EP4 was involved in the effect of PGE_2_ on c-jun in NSCLC [46].

Specific mitogen-activated protein kinase (MAPK) cascades control the activation of *fos* and *jun* family protooncogenes and their protein products (c-Jun and c-Fos) are known as the AP-1 family members. These “early response protooncogene” products dimerize to form the AP-1 transcription factor, a converging point that regulates the expression of genes involved in cell proliferation, differentiation, transformation, inflammation, pulmonary defense, and autoregulation of AP-1 gene transcription [46]. Early studies [47, 48] suggested that c-Jun had a role in early events in the pathogenesis of lung cancers because it was highly expressed in 31–50 % of patients with non- small cell cancers, and it was also upregulated in atypical bronchial epithelium. One study showed a transgenic mouse model directing conditional expression of the dominant-negative c-Jun mutant TAM67 in lung epithelial cells decreased tumor number and overall lung tumor burden in chemically induced mouse lung tumor models [49]. c-Jun is an important transcriptional activator of PDK1. Notably, expression of PDK1 is sufficient to restore tumor growth after c-Jun knockdown in melanoma cells, suggesting that PDK1 is an important mediator of c-Jun oncogenic activities [50]. Consistent with this, we found that PGE_2_ increased the expression of c-Jun and exogenous overexpression of c-Jun enhanced bronchial epithelial cells proliferation and expression of PDK1, whereas inhibition by PDK1 inhibitor or siRNA caused inhibitions of cell growth and expression of PDK1. This suggests that c-Jun is an upstream regulator of PDK1 signaling. Induction of c-Jun by PGE_2_ regulates the expression and activation of PDK1 and cell growth. It indicated c-Jun might play an important role in the potential mechanism of abnormal cell proliferation in brochial epithelial cells.

## Conclusion

In summary, our findings demonstrate that PGE_2_ stimulates human bronchial epithelial cell proliferation through activations of EP4-related signal and c-Jun, which, in turn, increases activation and expression of PDK1. This suggests that PDK1 and c-Jun might play an important role in human bronchial epithelial cells thereby influencing their growth.
